# Psychiatry on Twitter: Content Analysis of the Use of Psychiatric Terms in French

**DOI:** 10.2196/18539

**Published:** 2022-02-14

**Authors:** Sarah Delanys, Farah Benamara, Véronique Moriceau, François Olivier, Josiane Mothe

**Affiliations:** 1 Fédération Régionale de Recherche en Psychiatrie et santé mentale d’Occitanie Toulouse France; 2 Centre Hospitalier de Montauban Montauban France; 3 Institut de Recherche en Informatique de Toulouse Université de Toulouse Toulouse France

**Keywords:** social media analysis, psychiatric term use, social stigma, Twitter, social media, mental health

## Abstract

**Background:**

With the advent of digital technology and specifically user-generated contents in social media, new ways emerged for studying possible stigma of people in relation with mental health. Several pieces of work studied the discourse conveyed about psychiatric pathologies on Twitter considering mostly tweets in English and a limited number of psychiatric disorders terms. This paper proposes the first study to analyze the use of a wide range of psychiatric terms in tweets in French.

**Objective:**

Our aim is to study how generic, nosographic, and therapeutic psychiatric terms are used on Twitter in French. More specifically, our study has 3 complementary goals: (1) to analyze the types of psychiatric word use (medical, misuse, or irrelevant), (2) to analyze the polarity conveyed in the tweets that use these terms (positive, negative, or neural), and (3) to compare the frequency of these terms to those observed in related work (mainly in English).

**Methods:**

Our study was conducted on a corpus of tweets in French posted from January 1, 2016, to December 31, 2018, and collected using dedicated keywords. The corpus was manually annotated by clinical psychiatrists following a multilayer annotation scheme that includes the type of word use and the opinion orientation of the tweet. A qualitative analysis was performed to measure the reliability of the produced manual annotation, and then a quantitative analysis was performed considering mainly term frequency in each layer and exploring the interactions between them.

**Results:**

One of the first results is a resource as an annotated dataset. The initial dataset is composed of 22,579 tweets in French containing at least one of the selected psychiatric terms. From this set, experts in psychiatry randomly annotated 3040 tweets that corresponded to the resource resulting from our work. The second result is the analysis of the annotations showing that terms are misused in 45.33% (1378/3040) of the tweets and that their associated polarity is negative in 86.21% (1188/1378) of the cases. When considering the 3 types of term use, 52.14% (1585/3040) of the tweets are associated with a negative polarity. Misused terms related to psychotic disorders (721/1300, 55.46%) were more frequent to those related to depression (15/280, 5.4%).

**Conclusions:**

Some psychiatric terms are misused in the corpora we studied, which is consistent with the results reported in related work in other languages. Thanks to the great diversity of studied terms, this work highlighted a disparity in the representations and ways of using psychiatric terms. Moreover, our study is important to help psychiatrists to be aware of the term use in new communication media such as social networks that are widely used. This study has the huge advantage to be reproducible thanks to the framework and guidelines we produced so that the study could be renewed in order to analyze the evolution of term usage. While the newly build dataset is a valuable resource for other analytical studies, it could also serve to train machine learning algorithms to automatically identify stigma in social media.

## Introduction

### Stigma of Mental Disorders

Mental health stigma finds its roots in the history of psychiatry, in its connection to madness representations. Throughout history, the mentally ill patient has been given a pejorative label that induces social rejection. The term “stigma” comes from the ancient Greek “stitzein,” which means “to tattoo or mark with a red iron.” Jean-Yves Giordana [1], psychiatrist at the Nice hospital, rightly defines stigmatization as “a general attitude, a prejudicial one induced by low knowledge or ignorance of a situation or a state.”

Stigma and discriminatory behaviors have multiple negative impacts. Stigma in mental health leads the individual away from society, which often causes social isolation. Indeed, stigmatized people confront difficulties in their daily life such as integration into the professional world [1,2], access to housing [1], and interpersonal relationships [3]. Difficulties also concern the treatment itself, including delay in initial medical consultation, difficulty in accepting the illness, and tenuous therapeutic alliance, etc [1].

Many studies analyzing newspaper articles point out a major diversion in the use of psychiatric terms [4,5]. A French survey conducted by the L’Observatoire Société et Consommation [6] found that the French terms “schizophrène” (schizophrenic) and “schizophrénie” (schizophrenia) are particularly used in the context of violent news items and are often used metaphorically, which may lead to dangerousness, contradictory behavior, or negative connotation. Pignon et al [7], on the other hand, focused on the impact of the change of the terminology of bipolar disorder. The authors observed that substituting the term “manic-depressive psychosis” for the term “bipolar disorder” reduces stigma by disassociating this disorder from the representation of madness and dangerousness leading to the social exclusion classically associated with psychotic disorders.

With the rise of the internet and social media, it has become important to analyze how psychiatric terms are used by people in general to act effectively against stigmatization. Indeed, from the internet users’ point of view, Berry et al [8] showed that tweeting about mental health helps reduce isolation, fight stigmatization, and raise awareness of mental health by improving knowledge, promoting free expression, and strengthening coping and empowerment strategies.

In this paper, we focus on tweets in French as Twitter is one of the most used social media platforms in France [9], and the tweets are publicly available with some conditions.

### Twitter as a Resource to Analyze the Usage of Psychiatric Terms

More than 500 million tweets are posted daily in more than 40 languages [10]. In March 2019, Twitter had 321 million active users worldwide (at least one use per month) among which 10.3 million were in France [11], making Twitter third in popularity behind Facebook and YouTube, the other two most popular social networks, with 35 million and 19 million active users, respectively. The sociodemographic profile of Twitter users in France is more male, younger, and more educated than the general population. They are mainly students, with some managers and intellectual professions [12-14].

Twitter offers its users the opportunity to post short messages named “tweets” (140 characters maximum in our study although since we collected the data, the maximum length has doubled), making possible analyses of a large number of tweets in a short time. In addition, Twitter provides 1% of tweets posted each day worldwide, allowing free access to a large database accessible for various purposes including research.

Since 2014, many studies have addressed discourse content about psychiatry on Twitter, suggesting that social networks convey stigmatizing representations of mental health and people with mental health conditions. To our knowledge, existing studies deal only with English and Greek languages. Moreover, they focus on a limited number of psychiatric disorder terms such as depression, schizophrenia, and autism. Lachmar et al [15] created the hashtag #MDLL (#mydepressionlookslike) and analyzed 3225 tweets highlighting 7 topics when Twitter users talk about depression: dysfunctional thoughts, impact on daily life, social difficulties, hiding behind a mask, sadness and apathy, suicidal behaviors/ideas, and seeking support/help. Reavley et al [16] analyzed a corpus of tweets about schizophrenia and depression in English. This corpus was collected from the 1% database of Twitter using two keywords: #schizophrenia and #depression. They found that 5% of tweets related to schizophrenia convey stigmatizing remarks while less than 1% are related to depression. In addition, in their dataset, they found the polarity is mostly positive (65% of the tweets analyzed) when writing about depression while it is rather neutral (43%) for schizophrenia. Joseph et al [17] found that tweets containing the hashtag #schizophrenia convey a negative sentiment more frequently than tweets containing #diabetes (21% vs 12.6%, respectively). Similarly, Athanasopoulou et al [18] showed that tweets about schizophrenia in Greek tend to be more negative, medically inappropriate, sarcastic, and used in a nonmedical way than tweets about diabetes. Robinson et al [19] analyzed and compared messages about 5 psychiatric disorders (autism, depression, eating disorders, obsessive-compulsive disorder, and schizophrenia) and 5 physical diseases (AIDS, asthma, cancer, diabetes, and epilepsy). In their corpus, schizophrenia and HIV were the most stigmatized diseases. These diseases are perceived as dangerous and with an uncontrollable and unpredictable nature. The authors found more than 40% of stigmatizing tweets were about schizophrenia compared to less than 5% of those about depression. Finally, Alvarez-Mon et al [20] recently studied the use of the term “psychosis” and compared it to some medical terms from the field of somatic medicine (diabetes, HIV, Alzheimer disease, and breast cancer). The results showed a predominance of nonmedical content (33.3%) with a high frequency of misuse and pejorative opinion tone (36.2%) in the tweets related to psychosis compared to the tweets related to the physical diseases studied.

### Toward the First Analysis of Psychiatric Terms in French Tweets

As far as we know, this is the first study that proposes an in-depth analysis of psychiatric term usage in tweets in French. In particular, we propose the following:

- Analysis of a wide range of psychiatric terms going beyond a small set of nosographic terms. Our study considers a wide range of nosographic terms but also generic and therapeutic psychiatric terms.
- Multilayer annotation scheme that includes the type of word use (medical usage, misuse, or irrelevant usage) and the opinion orientation of the tweet (positive, negative, neutral, or mixed).
- New dataset of about 22,579 tweets containing the selected terms among which 3040 are manually annotated by clinical psychiatrists. The dataset will be made available to the research community.
- Qualitative analysis of the annotated data in terms of interannotator agreement along with quantitative analysis considering mainly term frequency in each layer and exploring the interactions between them.
- Comparison of our results to those obtained by analyzing tweets in English. Our results constitute a first important step toward an automatic detection of stigma in social media.

## Methods

### Objectives

The multidisciplinary study reported in this paper has been conducted by clinical psychiatrists and computer scientist experts in natural language processing and information extraction from social media. The main objective of the study is to analyze how psychiatric terms are used on Twitter, in particular whether they are used in a medical use. The other goal is to analyze the opinion polarity of these terms and thus highlight the main stereotypes they convey. Our assumption is that psychiatric terms are often misused and these misusages probably have a negative polarity.

### Tweet Collection

Our corpus is new and composed of tweets in French that contain selected terms relative to psychiatry. To guarantee a wide lexical convergence of the extracted tweets, we grouped terms according to 3 dimensions:

- Generic terms indicating different morphological variations of the French stem “psychiatr” (psychiatr) such as “psychiatrie” (psychiatry), “psychiatrique” (psychiatric) and “psychiatre” (psychiatrist)
- Nosographic terms relative to psychiatric disorders. Following the *Diagnostic and Statistical Manual of Mental Disorder* taxonomy [21] that classifies mental disorders in order to improve diagnoses, treatment, and research, we grouped terms in 5 main categories: schizophrenia spectrum and other psychotic disorders, bipolar and depressive disorders, autism spectrum disorder, anxiety disorders, and other disorders.
- Therapeutic terms relative to the most used drugs in the psychiatry field.

In each dimension, we selected a set of representative terms experts considered as the most important for this study. For each term, we also considered its slang versions, such as schizo for schizophrenia. We selected a total of 120 psychiatric terms (see Table 1 for examples and frequencies and Multimedia Appendix 1 for the detailed list).

Our dataset is composed of tweets collected via the OSIRIM platform that hosts a Twitter stream representing the 1% of global tweets since 2015, with a total of 73,345,245 tweets. From this collection, we selected tweets in French—using the tag provided by Twitter on tweets—that were posted from January 1, 2016, to December 31, 2018, and contain at least one psychiatric term from our list. After removing retweets and duplicates, we got at a total of 22,579 tweets (Table 2).

**Table 1:** Examples of terms for each dimension along with their frequencies and English translation (n=120).

Psychiatric terms			Example terms		terms, n
Generic			Psychiat-*		1
**Diagnostic**					31
	Schizophrenia spectrum	Psychose (*psychosis*), Psychotique (*psychotic*), Schizophrène (*schizophrenic*), Schizo (*schizo*)		6	
	Bipolar and depressive disorders	Maniaque (*manic*), Bipolaire (*bipolar*), Hypomaniaque (*hypomanic*)		7	
	Autism spectrum	Autisme (*autism*), Autiste (*autistic*)		2	
	Anxiety disorders	Phobie (*phobia*), TOC^a^ (*obsessive compulsive disorder*)		6	
	Other disorders	Hyperactif (hyperactive), borderline		10	
Therapeutic			Neuroleptique (*neuroleptic*), Xanax (*alprazolam*), Theralite (*lithium*)		88

^a^TOC: trouble obsessionnel compulsif.

**Table 2:** Number of tweets containing the selected terms (a tweet may contain several keywords).

Psychiatric terms		tweets, n
Generic		6993
**Diagnostic**		12,149
	Schizophrenia spectrum	1304
	Bipolar and depressive disorders	3500
	Autism spectrum	4389
	Anxiety disorders	5855
	Other disorders	101
Therapeutic		1853

### Annotation Guidelines

We designed an annotation guideline to analyze the use of the 120 selected psychiatric terms in tweets in French. To this end, two clinical psychiatrists first analyzed a small subset of 157 tweets randomly selected in order to define the annotation guidelines. These tweets were then removed from the initial collection and never used again in the study.

Our annotation scheme is multilayered and aims at answering 2 main questions: Do the psychiatric terms used in the tweet convey a medical use or not? What is the overall opinion given in the tweet? We detail each layer and illustrate them by example tweets extracted from our corpus. In these examples, psychiatric terms are in bold font. All examples are given in French along with their English translation. Note that translations may not perfectly reflect the initial writing, as tweets often use slang, abbreviations, and contain grammatical errors.

### Types of Term Use

We define three possible types of term use: medical use, misuse or irrelevant use.

Medical use corresponds to the medical definition of the term. The term is used to refer to a medical pathology or to the domain of psychiatry, as in Textbox 1.

Misuse occurs when a psychiatric term is used in a figurative or metaphoric way. These misuses often convey prejudices, stereotypes, or humor and thus make psychiatry commonplace and strengthen the stigma of psychiatry and people suffering from psychiatric disorders, as in Textbox 2.

Irrelevant use occurs when the tweet is not understandable (lack of context, link to a URL, advertising, etc) or not relevant to psychiatry (use of synonyms), as in Textbox 3.

**Examples of medical uses (psychiatric terms are in bold font).**

- *Tellement dégueulasse le **valium** en gouttes (Oral **valium** is so disgusting)*
- *Tout à l'heure g écouter une vidéo des voix qu'les **schizo** entendent dans leurs têtes g pas pu tenir + de 30sec g cru devenir folle (I listened to a video of voices heard by **schizophrenic** people I couldn’t hold more than 30 sec I thought I was going insane)*

**Examples of misuse (psychiatric terms are in bold font).**

- *Là j'suis en colère tu changes toutes les minutes, à croire que t'es **bipolaire**. (I’m angry you’re changing your mind every minute, I’d think you’re **bipolar**)*
- Tu viens d faire quoi sale **autiste** (What have you just done, you f*** **autistic**)

**Examples of irrelevant use (psychiatric terms are in bold font).**

- *qd t une **schizo** https://t.co/SB3Z1DR7cX (when a **schizophrenic** https://t.co/SB3Z1DR7cX)*
- ***Psychose**, C'est un peu vieux mais c'est trop cool (**Psycho**, it’s a little bit old but it’s so cool)*

### Overall Opinion of the Tweet

As usually defined in sentiment analysis [22], polarity or orientation indicates whether the opinion is positive, negative, or neutral. We consider these 3 possible values and also include mixed opinion to account for cases where the opinion can be positive and negative at the same time. We consider opinion orientation of the author at the tweet level regardless of whether the expressed opinion is related to a psychiatric term.

A tweet is annotated as having positive polarity when the writer expresses a positive personal opinion on facts, events, or on a quote (1); the general idea of the tweet is in favor of psychiatry (2); the writer defends the proper medical use of psychiatric terms regardless of their valence (3); or with the presence of positive terms or smileys (4), as in Textbox 4.

A tweet has a negative polarity when the writer expresses a negative personal opinion on facts, events, or on a quote (1); with the presence of terms that are basically negative (2); the tweet includes ironic or sarcastic comments (3); the tweet reports negative facts connected to psychiatry (4); the tweet contains a positive smiley linked to a negative content (5); the tweet marks a derogatory or insulting positioning (6); or the psychiatric term is used in the tweet to refer to an inconvenient situation or to a topic releasing a negative emotion (7), as in Textbox 5.

Mixed or neutral polarity orientation mainly covers cases where the opinion of the writer is not clearly expressed (1) or the writer’s opinion is mixed, both positive and negative (2), as in Textbox 6.

See Multimedia Appendix 2 for other examples of types of term use and polarity orientation.

**Examples of positive polarity (psychiatric terms are in bold font).**

1. *C'est trop top la **psychiatrie** tu vas t'éclater! (**Psychiatry** is so great, you’ll have so much fun!)*
2. *Mon Rdv **psychiatrie** de demain tombe à la perfection. Pour une fois je l'avoue, j'en ai grandement besoin. (Tomorrow is the perfect timing for my **psychiatric** appointment. To be honest, for once, I really need it)*
3. ***Bipolaire** c'est un vrai trouble **psychiatrique**, mesdames arrêtez de le mettre en TN vous n'êtes pas **bipolaires** vous êtes juste mal éduquées. (**Bipolar** disorder is a real **mental health** condition. Ladies, stop using this term as tweet name. You are not **bipolar**, you are just poorly-educated)*
4. *ça va mieux t'inquiète pas merci, j'ai pris 3 **Xanax** et ils commencent à faire effet (Feeling better, thanks, don’t worry. I took 3 **Xanax** tablets and it has started to work)*

**Examples of negative uses (psychiatric terms are in bold font).**

1. *La **psychiatrie** ça brise encore plus les gens. (**Psychiatry** breaks people down even more)*
2. *Il vend sa mère au diable se marie avec une chetana et Il finit en **psychiatrie**. Le pacte 666 l'a détruit. (He sells his mother to the devil, he gets married to a she-devil and he ends up in **psychiatric** hospital. He has been wiped out by pact 666)*
3. *La France est une terre d'asile... **psychiatrique** ! (France is a land of asylum… **psychiatric** asylum!)*
4. *Paris: la **psychiatre** vendait de faux certificats médicaux aux envahisseurs sans-papiers (Paris: **psychiatry** used to sell fake medical certificates to paperless invaders)*
5. *Les artistes finissent presque tous en hôpital **psychiatrique** / (Almost all artists end up in **psychiatric** hospital 
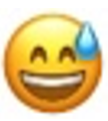
)*
6. *Selon une grosse conne **psychiatrique** le harcèlement d'activité est une loi de France (According to a **dumb lunatic** woman, harassing is a custom in France)*
7. *Et franchement les garçons radins c'est grave ma **phobie** (Sincerely, stingy boys are basically my greatest **phobia***

**Examples of mixed or neutral use (psychiatric terms are in bold font).**

1. *Croyez-vous qu'un **psychiatre** prendrait les médicaments qu'il prescrit (Do you think a **psychiatrist** would take the medicine he prescribes?)*
2. *La psychiatrie c'est cool, Faire ça dans un lieu de stage où ils te harcèlent jusqu'à la dernière heure de tout tout ton stage par contre moins. (**Psychiatry** is fun but throughout the internship they badger you, is less fun)*

### Annotation Procedure and Ethics

**Figure 1:**
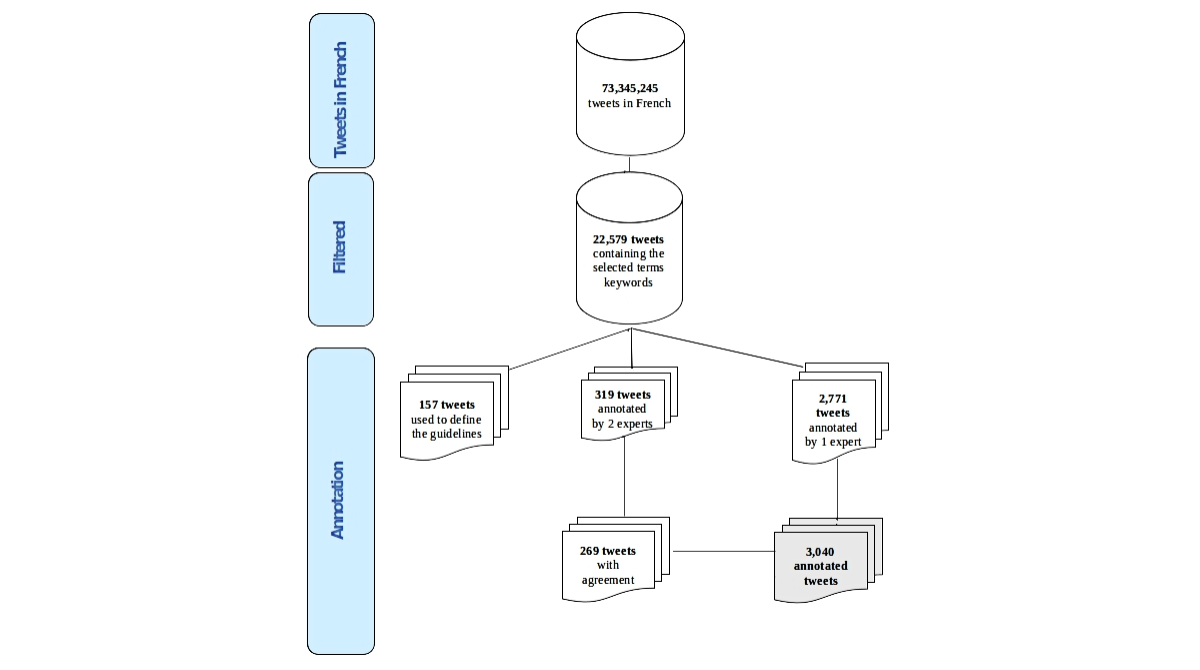
Annotation procedure.

Our data were manually annotated by two French native speakers, both clinical psychiatrists, using the Brat tool. We performed a 3-step annotation where an intermediate analysis of agreement and disagreement between annotators was completed. Annotators were first trained on 157 tweets that helped them better understand the task and adjust the annotation guidelines. Annotators were then asked to separately annotate 319 tweets (around 10% of the annotated corpus) so that interannotator agreements could be computed. Before moving to the real annotation, annotators were asked to discuss main cases of disagreement, which resulted in a set of 269 tweets. After adjudication, a total of 2771 tweets were manually annotated by one expert. In the end, our dataset is composed of 3040 tweets (269 + 2771) annotated according to our multilevel annotation scheme (Figure 1).

Regarding ethics, we did not request validation with the research ethics board since this study does not involve patients and does not use personal digital data. In addition, our data are composed of textual content from the public domain. Finally, as we will make the dataset publicly available to the research community and conform to the Twitter Developer Agreement and Policy that allows unlimited distribution of the numeric identification number of each tweet.

### Interannotator Agreement

Interannotator agreement allows assessment of the amount of agreement between annotators beyond chance and provides a measure of the reliability to the annotation guide. We used the Cohen kappa statistical measure defined as follows [23]: 
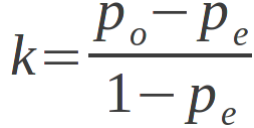


Where *p*_o_ and *p*_e_ are probabilities that correspond to the observed and the expected agreements, respectively. The latter probability measures the possible agreement by chance when each annotator randomly selects a given category. Kappa measure ranges from −1 to +1 where K ≤ 0 indicates no agreement, 0.6 ≤ K ≤ 0.8 a high agreement, and K = 1 a perfect agreement. We used Microsoft Excel to compute Cohen kappa from the contingency table of frequencies with the rows and columns indicating the categories (agreement frequencies are in the diagonal cells whereas disagreements are in the off-diagonal cells).

## Results

### Tweet Collection

We conducted a descriptive analysis that relies on the tweet collection we built . This does not require statistical tests.

From the initial collection of about 73 million tweets in French, 22,579 contain at least one term from our list of 120 terms. We observed that 25 terms out of 120 are not present in the dataset. They are mainly therapeutic terms such as international nonproprietary name of neuroleptics or antidepressants. The remaining 95 terms are diagnostic or generic terms referring to psychiatry.

From these 22,579 tweets, 3040 (13.46%) were manually annotated. Annotating tweets is time consuming and requires a high level of expertise in psychiatry. For this reason, we could annotate a limited number of tweets only. In future work, we will consider automatic annotation using supervised machine learning based on these examples, but this is out of the scope of this study.

Table 3 provides the overall frequency of annotated tweets for each dimension of psychiatric terms. Note that a tweet may contain several terms and hence the total is greater than 3040. We observe that tweets with diagnostic terms are the most frequent and that schizophrenia spectrum terms are dominant followed by generic and then bipolar disorders terms.

**Table 3:** Overall frequency of annotated tweets in each psychiatric term dimension.

Psychiatric terms		tweets, n
Total		3850
Generic		1086
**Diagnostic**		2604
	Schizophrenia	1300
	Bipolar/depressive	647
	Autism	232
	Anxiety	400
	Other disorders	25
Therapeutic		160

### Interannotator Agreement

In this section, we report on interannotator agreement on both the type of use and the overall opinion levels of the annotation scheme.

Kappa values were computed on the set of 319 tweets independently annotated by the two experts. For the types of term uses (that is to say medical use, misuse, or irrelevant use), we got a K=0.829 whereas for the opinion level (ie, positive, negative, or neutral), we got K=0.817. Interannotator agreement being very high (over 0.80), the guideline is considered reliable and the annotation reproducible.

We note that it is consistent with the percentage agreement, 84.3% (269/319).

### Analysis of Psychiatric Terms in the Annotated Dataset

Among the annotated tweets: 12.30% (374/3040) are annotated as irrelevant, 142.37% (1288/3040) as medical use 45.33% (1370/3040) as misuse. Concerning polarity, we could observe that 52.14% (1585/3040) are annotated as having a negative polarity, and 86.21% (1188/3040) of the tweets annotated as misuse are negative while 0.65% (9/3040) are positive. Furthermore, 19.02% (245/1288) of the tweets annotated as medical use have a positive polarity. It is interesting to note that most tweets annotated as medical use are neutral whereas tweets annotated as misuse are mostly negative (Table 4).

We can observe that misuses related to the spectrum of psychotic disorders are more frequent (721/1300, 55.46%) than those related to the spectrum of depression (15/280, 5.36%; Table 5).

Finally, term misuse with a positive polarity is rare and a lot of psychiatric terms such as “schizo” (schizophrenic), “bipolaire” (bipolar), and “autiste” (autistic) are used as insults. See the statistics in Multimedia Appendix 3.

In the remainder of this section, we provide a deeper inspection of each of our two annotation levels for both generic and diagnostic terms focusing on the 13 terms that are present in more than 50 tweets. We fixed this threshold to draw solid conclusions and reliable comparison with related studies. Therapeutic terms being rare in the annotated corpus, we did not include them in this detailed analysis.

**Table 4:** Distribution of annotated tweets according to the type of term use.

Psychiatric term	Types of term use, n							Irrelevant
	Medical			Misuse				
	Pos^a^	Neg^b^	Neut^c^	Pos	Neg	Neut		
Generic	109	269	376	0	252	17	63	
Schizophrenia	79	85	214	2	633	89	198	
Bipolar/depressive	125	102	196	7	117	47	53	
Autism	63	22	74	0	43	7	23	
Anxiety	26	51	94	0	176	20	33	
Other disorders	13	3	6	0	3	0	0	
Therapeutic	11	33	37	3	19	19	38	

^a^Pos: positive.

^b^Neg: negative.

^c^Neut: neutral.

**Table 5:** Frequency of term misuse according to the most frequent psychiatric terms in the annotated dataset.

Psychiatric terms	Frequency
Total	439
Schizo	70
Maniaque (manic)	65
Psychose (psychosis)	56
Phobie (phobia)	50
Autiste (autistic)	41
Bipolaire (bipolar)	41
Psychotique (psychotic)	40
Psychiatr-	25
Schizophrene (schizophrenic)	25
Schizophrenie (schizophrenia)	11
Depressif/ve (depressed)	7
Depression (depressive disorder)	5
Autisme (autism)	3

### Generic Terms

The terms “psychiatrie” (psychiatry), “psychiatrique” (psychiatric), and “psychiatre” (psychiatrist) have been collected using the stem “psychiatry.” These generic terms are mainly used in a medical use (754/1086, 69.43%), but only 14.5% (109/754) of these tweets have a positive polarity.

We observe that generic terms are often used with expressions like “ça relève de la psychiatrie” (It’s a matter of psychiatry) and “finir dans un hôpital psychiatrique” (to end up in a psychiatric hospital). The first expression occurs in 11 tweets and is related to a behavior or a person that is not understood, that seems different or out of the norm. In the same way, the second expression is used as a synonym of irrecoverable.

### Schizophrenia Spectrum and Other Psychotic Disorders

**Figure 2:**
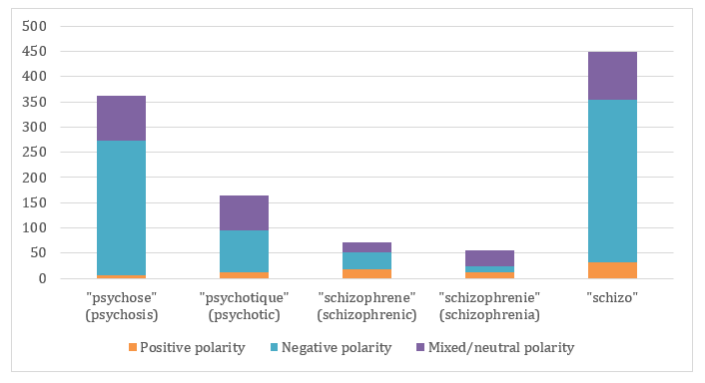
Polarity conveyed by any use according to the terms belonging to the spectrum of psychotic disorders.

We annotated all the tweets of the corpus that contain the terms “schizo,” “schizophrène” (schizophrenic), “schizophrénie” (schizophrenia), “psychose” (psycho), and “psychotique” (psychotic). Among the tweets containing these terms (Table 3), 48.69% (633/1300) are annotated as “misuse” with a negative polarity. All the terms from the spectrum of psychotic disorders have a negative polarity except for “schizophrenie” (schizophrenia), which has mainly a mixed or neutral polarity (Table 4). In particular, 70.1% (354/505) of the tweets containing “schizo” have been annotated as “misuse” versus only 25% (18/73) of the tweets containing “schizophrène.” On the other hand, “psychotique” (psychotic) is more misused (68/172, 39.5%) than “schizophrène” (18/73, 24.66%). In the same way, “psychose” (psycho) is frequently misused (278/494, 56.3%), meaning an excessive fear, a collective terror maintained by a stressful environment or by media. It is often related to violent events, terrorist attacks, or in a context of political tensions. It is more misused than “schizophrénie” (shizophrenia) (6/56, 11%; see Figure 2).

### Depressive and Bipolar Disorders

In the domain of depression, all tweets containing the terms “depression” (nervous breakdown) and “dépressif/ive” (depressive) have been annotated, and 5.4% (15/280) are annotated as “misuse.”

**Figure 3:**
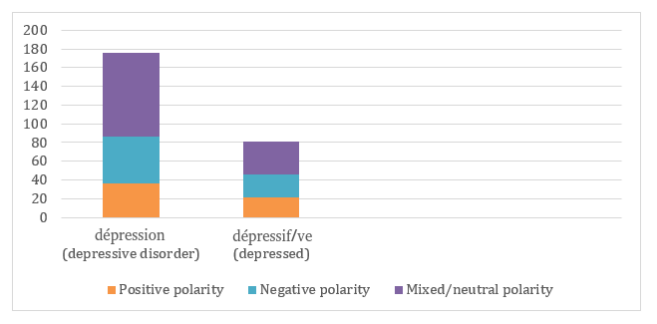
Polarity conveyed by any use according to the depression spectrum terms in the annotated dataset.

From Figure 3, we see that 26.2% (50/191) to 28% (25/89) of the tweets only (depending on the keyword) have a negative polarity. When depression is concerned, tweets could be sorted into 5 categories: personal testimony or experience, recourse to care, defense of medically correct use, qualification of a state of mind, and misused to evoke something other than psychological behavior.

When analyzing the distribution of terms relative to bipolar disorders (mainly the term “bipolaire” [bipolar]), 40.6% (112/276) of the tweets are annotated as “misuse” and 24.2% (58/240) of those annotated as “medical use” have a positive polarity. These tweets often describe a person who changes her or his mind or emotions (going from tears to laughter) or who is difficult to understand (as in “Là j’suis en colère tu changes toutes les minutes, à croire que t’es bipolaire” [I’m angry you’re changing your mind every minute, it seems like you’re bipolar]). This term is sometimes related to objects, animals, weather, or political parties that have changing behaviors or contradict themselves.

Finally, 65% (34/52) of the tweets containing the term “maniaque” (maniac) are annotated as “misuse,” the term being most often a synonym of obsession. This is one of the few terms being misused but having a positive polarity (4/34, 12%).

### Anxiety Disorders

The term “phobie” (phobia) is the most frequent specific term in our corpus (5494/22,579, 24.33%) and is related to something that is hated or causes anger. Among the annotated tweets containing this term, 50.1% (180/359) are annotated as “misuse” and have mostly a negative polarity (163/180, 90.56%).

### Autism Spectrum Disorder

**Figure 4:**
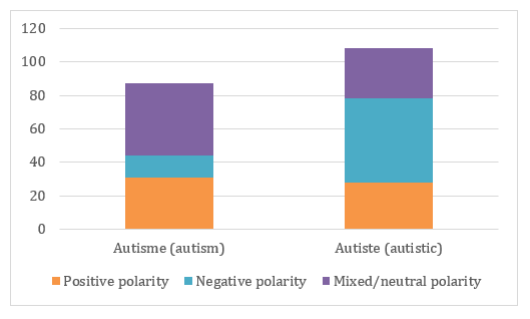
Polarity conveyed by any use according to the autism spectrum disorder terms in the annotated dataset.

In the domain of autism, we found that 40.9% (47/115) of the tweets containing the term “autiste” (autistic) are annotated as “misuse” and 43.5% (50/115) have a negative polarity. This is not the case for the term “autism” (autism), which is annotated as “medical use” in 91% (84/92) of the tweets and has a negative polarity in only 14% (13/92) of the tweets. The term “autiste” seems to be close to an insult meaning idiot or referring to a person with less adaptation and strange behaviors. On the contrary, the term “autisme” has most often a positive polarity: we noticed that a lot of tweets defend the medical use of this term by conveying information, testimonials from autistic people and families or references to articles about autism. These messages allow one to fight against prejudices (Figure 4).

## Discussion

### Principal Findings

The main goal of this study was to analyze how psychiatric terms are used on Twitter. A descriptive analysis of our annotated corpus shows that terms from the psychiatric domain are often misused (1378/3040, 45.33%) and that most of the tweets about psychiatry convey a negative polarity (1585/3040, 52.14%).

There are some differences in psychiatric term distribution in our corpus. Indeed, there are far fewer tweets about depression than psychosis. This difference may also be due to the fact that there are many more terms related to psychiatry than to depression and thus potentially more tweets. The most frequent psychiatric terms in our corpus are “phobie,” “bipolaire,” and “autiste,” probably meaning that they now belong to everyday language.

### Comparison With Related Work in Social Media

Our analysis reveals that depression is less prone to stigmatization, in the sense of term misuse, than schizophrenia. These results confirm the existence of stigmatization and negative prejudices related to psychotic disorders. In particular, thus, the terms “dépression” and “autisme” are less prone to misuse than the terms “ psychose” and “psychotique” as well as “schizophrène” and “schizophrénie.” This trend toward stigmatization is consistent with the results of previous work in English social networks [16,19]. For example, Robinson et al [19] found stigmatizing behaviors in less than 6% of tweets about depression and autism versus more than 40% of tweets about schizophrenia. Regarding polarity, Joseph et al [17] found that 33% and 21.1% of tweets containing the words “schizophrenic” and “schizophrenia,” respectively, convey a negative opinion, which is consistent with our results of 47% (34/73) and 18% (10/56), respectively. On the other hand, the term “depression” in our study is less often associated with a positive polarity (36/191, 18.9%) versus 65% according to Reavley et al [16]). This difference may reflect the lack of consensus in how to define the positive polarity but also the variability in the sample size or way the tweets were collected.

### Limitations

Our results cannot be directly generalized to the discourse conveyed on Twitter or to the representations conveyed in the general population for the following reasons. First, the Twitter user community is not representative of the general population. Indeed, no sociodemographic data were collected to describe the characteristics of the tweet authors in our corpus. Second, the link between thought and written discourse remains complex, which is why it is impossible to extrapolate the ideas conveyed on Twitter to social representations. Third, the method we used to collect tweets also contributes to the difficulty of generalizing our results since Twitter does not provide information concerning the representativeness of the 1% of tweets compared to all the tweets posted daily. Thus, the results of this study apply to our corpus of tweets but nothing can be said on their generalization, especially since our annotated dataset is relatively small. An alternative way to collect tweets could have been to use the Twitter Filter Realtime Streaming API, although because of limitations, we are not fully sure a replication of this can be guaranteed to collect the same tweets (eg, removed tweets), while since we are storing them, we can guarantee replication.

Another limitation concerns the manual annotation itself as the dataset is naturally biased toward annotators’ subjectivity mainly because tweets are short and the lack of context forces annotators to rely on cultural and background knowledge to better understand the tweet content. This is particularly true in case of irony and humor. Overall, this is a more general problem when manually building linguistic resources [24], and the reliable interannotator agreement we obtained (0.829 for the types of use and 0.817 for the opinion level) guarantees a good quality of the resource produced in this study.

### Conclusions

The analysis of the messages posted on Twitter in French in this pilot descriptive study highlights the existence of a misuse of most of the psychiatric terms studied in our corpus of tweets and the preponderance of a negative polarity conveyed by tweets when talking about psychiatry. It is important to mention that this work, despite some limitations, is part of an international research landscape that is expanding in recent years.

We believe it is necessary to pursue this research on digital social networks in order to improve the quality of discourse analysis and to work toward a better representativeness. Such study is also useful to evaluate the impact of antistigma campaigns on the content of social networks. To this end, future research could focus on a larger corpus of tweets and/or to other popular social networks. Another future direction is the automatic detection of stigma relying on natural language processing techniques. The annotated dataset that we built can then serve as a valuable source to train machine learning techniques to jointly identify the type of use and the opinion conveyed by each tweet.

## Appendix

Multimedia Appendix 1 List of terms used in our study grouped in three dimensions: generic, diagnostic and therapeutic.

Multimedia Appendix 2 Additional tables.

Multimedia Appendix 3 Term frequencies.
