# Supplementary material for: Psychiatry on Twitter: Content Analysis of the Use of Psychiatric Terms in French
Source: JMIR Form Res. 2022 Feb 14;6(2):e18539. doi: 10.2196/18539 (PMC8887636; doi:10.2196/18539)
Supplement: Multimedia Appendix 1 [file formative_v6i2e18539_app1.docx]

Multimedia Appendix 1. List of terms used in our study grouped in three dimensions: generic, diagnostic and therapeutic.

| **General terms** | **Psychiatric disorders** | **Therapeutics** |
| --- | --- | --- |
| - psychiatr- | **Psychotic disorders** | **Drugs** |
|  | psychose  psychotique  schizophrene  schizophrenie  schizo  schizoaffectif | antidepresseur  prozac/fluoxetine  deroxat/paroxetine  zoloft/sertraline  effexor/venlaflaxine  duloxetine/cymbalta  anafranil/clomipramine  athymil/mianserine  norset/mirtazapine  seroplex/escitalopram  seropram/citalopram  laroxyl/amitriptyline  brintellix/vortioxetine  anxiolytique  seresta/oxazepam  xanax/alprazolam  temesta/lorazepam  lexomil/bromazepam  valium/diazepam  lysanxia/prazepam  tranxene/clorazepate  neuroleptique  antipsychotique  haldol/haloperidol  zyprexa/olanzapine  abilify/aripiprazole  leponex/clozapine  xeroquel/quetiapine  risperdal/risperidone  nozinan/levomepromazine  loxapac/loxapine  tercian/cyamemazine  solian/amisulpride  clopixol/zuclopenthixol  xeplion  maintena  consta  trevicta  decanoas  fluanxol  modecate  piportil  thymoregulateur  teralithe/lithium  lamictal/lamotrigine  depakote/depamide/acide valproique  tegretol/carbamazepine |
|  | **Mood disorders** |  |
|  | depressi-  melancoli-  maniaque  hypomaniaque  maniaco-depressi-  PMD  bipolaire |  |
|  | **Anxiety disorders** |  |
|  | phobie  obsessionnel  agoraphobie  TOC  PTSD  stress post-traumatique |  |
|  | **Other disorders** |  |
|  | TCA  anorexi-  boulimi-  borderline  etat limite  TDAH  hyperactif  autisme  autiste  TED  TSA  asperger |  |
|  |  | **other therapeutics** |
|  |  | sismotherapie  electroconvulsivotherapie  electrochoc  electronarcose/narcose |
