# Supplementary material for: Psychiatry on Twitter: Content Analysis of the Use of Psychiatric Terms in French
Source: JMIR Form Res. 2022 Feb 14;6(2):e18539. doi: 10.2196/18539 (PMC8887636; doi:10.2196/18539)
Supplement: Multimedia Appendix 2 [file formative_v6i2e18539_app2.docx]

## Multimedia Appendix 2. Additional tables.

Table S1. Examples of tweets and their corresponding category.

| **Medical use** | **Misuse** | **Irrelevant use** |
| --- | --- | --- |
| *« Tellement dégueulasse le valium en gouttes »*  *« Oral valium is so disgusting »*  *« Je sors de l'hôpital psychiatrique, plus démolie que jamais. Force à toi. »*  *« I just left the psychiatric hospital even more downcast. May the force be with you »*  *« 17 mars : Journée mondiale de la Schizophrénie. La schizophrénie est une maladie du cerveau qui affecte la pensée, les sentiments et les émotions. »*  *« March 17: World Schizophrenia Day. Schizophrenia is a mental illness that affects a person’s thinking, feelings and emotions »*  *« Tout à l'heure g écouter une vidéo des voix qu'les schizo entendent dans leurs têtes g pas pu tenir + de 30sec g cru devenir folle »*  *« I just listened to a video recording voices heard by schizophrenic people. I could not hold more than 30 sec. I thought I was going insane »*  *« Sensibiliser tous les acteurs aux spécificités de la prise en charge de l’autisme et aux dispositifs existants #AvecBLM »*  *« To raise awareness amongst all actors on the specifics of managing autism and the existing devices #AvecBLM »*  *« 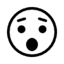 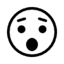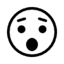 Un Français avoue le meurtre de quatre membres de sa famille - https://t.co/3Hh4Iq8nQD #Psychiatrie #SantéMentale »*  «  *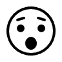 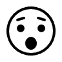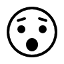 A French man confesses the killing of four people of his family* - *https://t.co/3Hh4Iq8nQD #Psychiatry #Mentalhealth »* | *« Y vont me rendre ouf dans ce lycée c'est sur. L'année pro c'est pas en BTS que je vais aller c'est en psychiatrie pour meurtre »*  *« They gonna drive me crazy for sure in this high school. Next year I won’t be in a BTS class but in a psychiatric hospital after committing murder»*  *« L'international socialisme en pleine régression au stade fécal Aux frontières de l'internement psychiatrique #PS »*  *« International socialism decreases to a fecal stage. At the boundaries of psychiatric internment #PS »*  *« Lui, il est bon pour l'asile psychiatrique ! »*  *« On a un peu l'impression que les réseaux sociaux sont un hôpital psychiatrique à ciel ouvert »*  *« He’s done for psychiatric asylum »*  *« It feels like social media are an open-air psychiatric hospital »*  *« Les vêtements couleurs léopard qui redeviennent à la mode ma + grosse phobie ce truc »*  *« Clothing with leopard pattern is back into style, this stuff is my greatest phobia »*  *« En ce moment je sais pas pourquoi mais je deviens vraiment schizophrène à gueuler pour rien »*  *« I don’t know why but these days I feel like I’m schizophrenic when shouting for no reason »*  *« Jsuis tellement maniaque que je me sens pas bien qd je termine pas une série »*  *« I’m so maniac that I don’t feel well when I don’t finish a series”*  *« C'est quand même affolant le nombre « d'élus » à des postes relativement importants, qui relèvent de la psychiatrie lourde »*  *« It is quite alarming that so many elected representatives holding a relatively important position are to receive psychiatric care. »*  *« Là j'suis en colère tu changes toutes les minutes, à croire que t'es bipolaire. »*  *« I am angry, you’re changing your mind every minute, it seems like you’re bipolar »*  *« Tu viens d faire quoi sale autiste »*  *«What have you just done, you f*** autistic»* | **Tweets with poor content preventing the analysis of use** |
|  |  | *« Basisarts Psychiatrie (Assen) https://t.co/WlVOshngli #careerjet Vacature Assen »*  *« qd t une schizo https://t.co/SB3Z1DR7cX »*  *« skizo forcenés de harcelement de petes psychiatrique »* |
|  |  | **Tweets referring to commercial advertising and employment opportunities** |
|  |  | *« Nouvelle offre d'emploi sur notre site : Médecin Psychiatre (H/F) - Stages et internat de médecine, Moins de 5... https://t.co/DrFauStHZ8 »*  *« New employment opportunity on our website: Psychiatrist (M/F) – Internships and Internship in Medicine, less than 5... https://t.co/DrFauStHZ8 »*  *« @IvanOGodfroid Psychiatre littéraire. Tous ses livres sont à 1 € symbolique, Découvrez les ici : https://t.co/ketYcd1yVP ou @Kobo »*  *« @IvanOGodfroid Psychiatrist and literature specialist. All his books are sold for a symbolic euro Discover them here : https://t.co/ketYcd1yVP or @Kobo »* |
|  |  | **Tweets not related to psychiatry** |
|  |  | *« j'me suis crue dans un film aussi à asperger tout le monde avec du champ ou quoi »*  *« I though I was in a movie with the Champagne shower thing »*  *« Le marché du #lithium s’envole, un risque pour notre approvisionnement ? https://t.co/FIwFJI009v #transitionénergétique #enr #écomobilité #solaire #écomobilité #cobalt »*  *« #Lithium market is soaring, what’s the risk for procurement?*  *https://t.co/FIwFJI009v #energytransition #enr #ecomobility #solar #ecomobility #cobalt »*  *« Psychose, C'est un peu vieux mais c'est trop cool » le film de Hitchcock*  *« Psycho, a bit old but so cool » Hitchcock’s movie* |
|  |  | **Tweets referring to a URL with poor textual content** |
|  |  | *« Phobie scolaire https://t.co/NrqcPL3Y5C »*  *« School phobia » https://t.co/NrqcPL3Y5C »* |

Table S2. Examples of tweets classified according to their polarity.

| **Positive opinion** | **Negative opinion** | **Mixed/Neutral** |
| --- | --- | --- |
| **The writer expresses a positive personal opinion on facts, events or on a quote** | **The writer expresses a negative personal opinion on facts, events or on a quote** | **The opinion of the writer is not clearly expressed** |
| *« C'est trop top la psychiatrie tu vas t'éclater ! »*  *« Psychiatry is so great, you’ll have so much fun! »* | *“La psychiatrie ça brise encore plus les gens.”*  *“Psychiatry breaks people down even more”*  *« Cette femme-là devrait être soit en prison soit dans un hôpital psychiatrique »*  *« This woman should either be in jail or in psychiatric hospital »* | *« Croyez-vous qu'un psychiatre prendrait les médicaments qu'il prescrit »*  *« Do you think a psychiatrist would take the medicine he prescribes? »*  *« Lundi j'ai été mise dans la section psychiatrique d'un hôpital. Cette section est pour les personnes entre 10 et 15 ans. »*  *« On Monday, I was admitted to psychiatric ward. This department is for people aged 10 to 15 »*  *« Tu as une phobie ? Si oui laquelle ? »*  *« Do you have a phobia? What is it? »* |
| **The general idea of the tweet is in favour of psychiatry** | **The valence of the terms used in the tweet are basically negative** | **The writer’s opinion is mixed, both positive and negative** |
| *« Mon Rdv psychiatre de demain tombe à la perfection. Pour une fois je l'avoue, j'en ai grandement besoin. »*  *« Tomorrow is the perfect timing for my psychiatric appointment. To be honest, for once, I really need it »*  *« Phobie, rééducation, paraplégie : quand la réalité virtuelle soigne »*  *« Phobia, rehabilitation, paraplegia : healing with virtual reality »* | *« Il vend sa mère au diable se marie avec une chetana et Il finit en psychiatrie. Le pacte 666 l'a détruit. »*  *« He sells his mother to the devil, he gets married to a she-devil and he ends up in psychiatric hospital. He has been wiped out by pact 666 »* | *« La psychiatrie c'est cool, Faire ça dans un lieu de stage où ils te harcèlent jusqu'à la dernière heure de tout tout ton stage par contre moins. »*  *« Psychiatry is fun but throughout the internship they harass you, less fun »* |
| **The writer defends the proper medical use of psychiatric terms regardless of their valence** | **The tweet includes ironic or sarcastic comments** |  |
| *« Faut arrêter d'utiliser bipolaire à tout-va hein »*  *« Stop using bipolar randomly, will you »*  *« Bipolaire c'est un vrai trouble psychiatrique, mesdames arrêtez de le mettre en TN vous n'êtes pas bipolaires vous êtes juste mal éduquées. »*  *« Bipolar disorder is a real mental health condition. Ladies, stop using this term as tweet*  *name. You are not bipolar, you are just poorly-educated »*  *« Bipolaire c'est PAS lunatique, anxieux c'est PAS être stressé et être triste c'est PAS être dépressif et j'en passe »*  *« To be bipolar is NOT being volatile, to be anxious is NOT being stressed, to be sad is NOT being depressed, and so on »* | *« La France est une terre d'asile... psychiatrique ! »*  *« France is a land of asylum… psychiatric asylum! »* |  |
| **The valence of the terms or smileys is mainly positive** | **The tweet reports negative facts connected to psychiatry** |  |
| *« ça va mieux t'inquiète pas merci, j'ai pris 3 Xanax et ils commencent à faire effet »*  *« Feeling better, thanks, don’t worry. I took 3 Xanax tablets and it has started to work »*  *« Je recommence à avoir les symptômes du trouble borderline comme y a trois ans  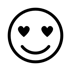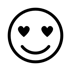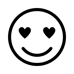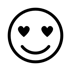 »*  *« I am having borderline disorder symptoms again, like three years ago 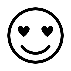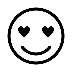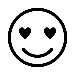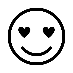 »* | *« Paris : la psychiatre vendait de faux certificats médicaux aux envahisseurs sans-papiers »*  *« Paris: the psychiatrist used to sell fake medical certificates to paperless invaders*  *« La psychiatrie toujours en crise @HopitaldeNiort »*  *« Psychiatry still in crisis @HopitaldeNiort »* |  |
|  | **The tweet contains a positive smiley linked to a negative content** |  |
|  | *« Les artistes finissent presque tous en hôpital psychiatrique 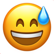 »*  *« Almost all artists end up in psychiatric hospital 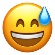 »* |  |
|  | **The tweet marks a derogatory or insulting positioning** |  |
|  | *« Selon une grosse conne psychiatrique le harcèlement d'activité est une loi de France »*  *« According to a dumb lunatic woman, harassing is a custom in France »*  *« Quand tu dis un truc et son contraire en 140 caractères #MercatOM #Schizo »*  *« When you say something and its opposite with 140 characters*  *#MercatOM #Schizo »* |  |
|  | **The psychiatric term is used in the tweet to refer to an inconvenient situation or to a topic releasing a negative emotion** |  |
|  | *« Et franchement les garçons radins c'est grave ma phobie »*  *« Sincerely, stingy boys are basically my greatest phobia »* |  |
