# Supplementary material for: Psychiatry on Twitter: Content Analysis of the Use of Psychiatric Terms in French
Source: JMIR Form Res. 2022 Feb 14;6(2):e18539. doi: 10.2196/18539 (PMC8887636; doi:10.2196/18539)
Supplement: Multimedia Appendix 3 [file formative_v6i2e18539_app3.docx]

## Multimedia Appendix 3. Term frequencies.

The most frequent terms along with the number of annotated tweets containing these words, the percentage of tweets with misuse, and the percentage of tweets conveying negative opinions, regardless the type of term use.
